# Extracellular Citrate Is a Trojan Horse for Cancer Cells

**DOI:** 10.3389/fmolb.2020.593866

**Published:** 2020-11-12

**Authors:** Agata Petillo, Vittorio Abruzzese, Prashant Koshal, Angela Ostuni, Faustino Bisaccia

**Affiliations:** Laboratory of Cell Biochemistry, Department of Sciences, University of Basilicata, Potenza, Italy

**Keywords:** citrate, HepG2, IHH, ATP citrate lyase, histone acetylation, cancer, epigenetics

## Abstract

The first intermediate in the mitochondrial tricarboxylic acid (TCA) cycle is citrate, which is essential and acts as a metabolic regulator for glycolysis, TCA cycle, gluconeogenesis, and fatty acid synthesis. Within the cytosol, citrate is cleaved by ATP citrate lyase (ACLY) into oxaloacetate (OAA) and acetyl-CoA; OAA can be used for neoglucogenesis or in the TCA cycle, while acetyl-CoA is the precursor of some biosynthetic processes, including the synthesis of fatty acids. Accumulating evidence suggests that citrate is involved in numerous physiological and pathophysiological processes such as inflammation, insulin secretion, neurological disorders, and cancer. Considering the crucial role of citrate to supply the acetyl-CoA pool for fatty acid synthesis and histone acetylation in tumors, in this study we evaluated the effect of citrate added to the growth medium on lipid deposition and histone H4 acetylation in hepatoma cells (HepG2). At low concentration, citrate increased both histone H4 acetylation and lipid deposition; at high concentration, citrate inhibited both, thus suggesting a crucial role of acetyl-CoA availability, which prompted us to investigate the effect of citrate on ACLY. In HepG2 cells, the expression of ACLY is correlated with histone acetylation, which, in turn, depends on citrate concentration. A decrease in H4 acetylation was also observed when citrate was added at a high concentration to immortalized human hepatic cells, whereas ACLY expression was unaffected, indicating a lack of control by histone acetylation. Considering the strong demand for acetyl-CoA but not for OAA in tumor cells, the exogenous citrate would behave like a trojan horse that carries OAA inside the cells and reduces ACLY expression and cellular metabolism. In addition, this study confirmed the already reported dual role of citrate both as a promoter of cell proliferation (at lower concentrations) and as an anticancer agent (at higher concentrations), providing useful tips on the use of citrate for the treatment of tumors.

## Introduction

Metabolism is a fundamental biological process in all living organisms for various cellular activities, such as maintaining homeostasis and producing functional energy, building blocks, enzymatic cofactors, and signaling molecules. However, these metabolic processes are also associated with the generation of several metabolites and in the activation of many enzymes, which are involved in the regulation of gene expression, immunoreactions, cellular apoptosis, and cancer progression (van der Knaap and Verrijzer, 2016; He et al., 2017). It is widely accepted that cancer cells alter their metabolic pathways to generate more fatty acids from lipogenesis to meet the increasing energy demand for rapid cell division and propagation (De Berardinis and Chandel, 2016). The reprogramming of metabolic pathways in cancer cells alters the expression of certain genes and directly controls glycolysis, lipogenesis, and nucleotide synthesis, which have the potential to be considered as prognostic markers and therapeutic targets in the development of new anti-cancerous agents (Fritz and Fajas, 2010; Furuta et al., 2010; Liao, 2017; Liu et al., 2019).

One classical example of a reprogrammed metabolic pathway in cancer is the Warburg effect or aerobic glycolysis, which is characterized by an increased rate of glycolysis, resulting in a high production of lactic acid despite having sufficient oxygen (Liberti and Locasale, 2016; Burns and Manda, 2017). However, some cancer cells are also able to switch their metabolism between glycolysis and mitochondrial oxidative phosphorylation (Roth et al., 2020). Reprogramming of glucose metabolism and targeting altered metabolic pathways related with glucose metabolism may contribute to designing novel treatment strategies for improving the efficacy of cancer therapy (Luengo et al., 2017; Lin et al., 2020).

Citrate is an essential intermediate in the tricarboxylic acid (TCA) cycle which comes from the metabolism of glucose and glutamine (Icard et al., 2012). It is released into the cytoplasm through the mitochondrial citrate transporter SLC25A1, a member of the solute carrier transporter family that operates as a citrate/malate exchanger (Bisaccia et al., 1989) and, if added to the culture medium (exogenous citrate), enters the cells through the sodium-dependent transporter SLC13A5 (Costello and Franklin, 2016). Both exogenously added and mitochondrially produced (endogenous) citrate are cleaved into acetyl-CoA and oxaloacetate (OAA) in the cytosol by ATP citrate lyase (ACLY). In normal cells, when citrate is produced by TCA cycle and ATP levels are high, citrate exerts a negative feedback on glycolysis and TCA cycle itself. In the cytosol, ACLY provides acetyl-CoA, which sustains some biosynthetic processes, including lipid synthesis and acetylation of both histone and non-histone proteins. In cancer cells, to prevent the suppression of glycolysis, citrate is rapidly converted into OAA and acetyl-CoA by ACLY, which is overexpressed (Zaidi et al., 2012; Khwairakpam et al., 2015; Khwairakpam et al., 2020). The conversion of OAA into malate by malate dehydrogenase produces NAD^+^ to sustain glycolysis (Gatenby and Gillies, 2004). Besides being a source of acetyl-CoA to sustain lipid biosynthesis, citrate is a key substrate for the activity of histone transacetylases, which, together with histone deacetylases, are responsible for some epigenetic modifications (Wellen et al., 2009).

Citrate acts as a regulator of multiple physiological and pathophysiological processes such as insulin secretion (Mohammad et al., 2007), inflammation (Williams and O’Neill, 2018), neurological disorders (Abdel-Salam et al., 2014), and cancer. There is growing evidence suggesting that the anti-tumor effect of exogenous citrate may be due to the regulation of some key enzymes of glycolysis, TCA cycle, gluconeogenesis, and fatty acid synthesis (Pastorino and Hoek, 2008; Ren et al., 2017). *In vitro* and *in vivo* studies showed that citrate at a high concentration inhibited the proliferation of several cancer cell types by inducing mitochondria-mediated apoptosis (Wang et al., 2018; Caiazza et al., 2019). Nevertheless, some citrate-resistant cells are able to adapt to high citrate concentrations. Interestingly, it has been observed that, at physiological concentrations (200 μmol/L), some tumor cell lines take up larger amounts of citrate than the normal cells and that gluconate, by inhibiting the citrate plasma membrane carrier, reduced the growth of human pancreatic tumors implanted subcutaneously in mice (Mycielska et al., 2018).

Considering the crucial role of citrate to supply the acetyl-CoA pool for fatty acid synthesis and histone acetylation in tumors (Wellen et al., 2009; Lee et al., 2014; Khwairakpam et al., 2020), in the present study, we assessed the effect of exogenous citrate supplementation on both ACLY expression and histone H4 acetylation in hepatoma cells (HepG2). All the experiments were performed at high and low glucose concentrations in order to verify that the observed effects are attributable to the involvement of citrate into glucose metabolism as well as to make a quantitative assessment. Moreover, this approach allowed to exclude possible non-specific effects due to the chelating activity of citrate (Sul et al., 2016). We sought to determine the effect of citrate on human immortalized cells (IHH) to understand the specificity of action of exogenous citrate on tumor vs normal cell types.

Interestingly, all the results also allowed to analyze the contrasting and dose-dependent effects of externally administered citrate to cancer cells and to suggest a molecular mechanism underlying the antitumor effect of citrate when used at a high concentration.

## Materials and Methods

### Cell Culture and Treatments

Human hepatocellular carcinoma cell line (HepG2) was maintained in Dulbecco’s modified Eagle’s medium (DMEM) containing 25 or 5 mM glucose, supplemented with 10% fetal bovine serum (FBS), 2 mM L-glutamine, penicillin (100 U/ml), and streptomycin (100 mg/ml), and immortalized human hepatocyte (IHH) cells were maintained in DMEM F-12 supplemented with 10% FBS, 1% of 100 IU/ml penicillin, 100 μg/ml streptomycin, 1 μM dexamethasone, and 10^–12^ M insulin. All the cell lines were cultured at 37°C and 5% CO_2_ in a humidified incubator. The HepG2 and IHH cells were seeded on culture plates and treated for 24 h with sodium citrate and Trichostatin A (TSA), which is an inhibitor of histone deacetylase (HDAC) that prevents the removal of acetyl groups from lysine residues on histone tails. Sodium citrate was dissolved in distilled water at 770 mg/ml; TSA was dissolved in dimethyl sulfoxide (DMSO) at 2 mg/ml as stock solution, which was then diluted with cultured medium to the desired concentrations (Salvia et al., 2017). The final concentration of DMSO did not exceed 0.25% v/v. The control cells were treated at the same final percentage of DMSO. All compounds were purchased from Sigma-Aldrich (unless otherwise indicated).

### Viability Assay

Cells were seeded at a density of 1.5 × 10^3^/well in 96-well plates and incubated with various concentrations of sodium citrate (1, 5, 10, and 20 mM) for 24 h (except the untreated control). The cells were treated with 0.75 mg/ml of [3-(4,5-dimethyl thiazol-2yl)-2,5-diphenyl tetrazolium bromide] (MTT) solution in DMEM for 4 h at 37°C. The solution was subsequently removed, and the cells were lysed using a solubilization solution (1:1 DMSO/isopropanol with 1% of Triton X-100). The solubilized formazan product was spectrophotometrically quantified at 570 nm using a microplate reader (Multiskan^TM^ GO Microplate Spectrophotometer, Thermo Fisher Scientific). The results were presented as percentage of the control, defined as 100% of cell viability. Each test was repeated three times in triplicate.

### Western Blot Analysis

Western blot analysis was performed as previously reported (Miglionico et al., 2017), with some modification. Cells were lysed in Laemmli sample buffer (4% SDS, 20% glycerol, 200 mM DTT, 0.01% bromophenol blue, and 0.1 M Tris–HCl, pH 6.8) by sonication with Bandelin Sonopuls Ultrasonic Homogenizers. Finally, the proteins were resolved on 12% SDS-PAGE gels. After electroblotting on nitrocellulose membrane (AmershamProtran, GE Healthcare Life Sciences), membranes were blocked for 1 h with 5% non-fat milk in PBS-T, pH 7.4, and incubated overnight at 4°C with specific primary antibodies: 1:10,000 anti-β-actin, 1:400 anti-ACLY (Invitrogen), and 1:400 anti-Ac-H4 histone (Santa Cruz Biotechnology), diluted in PBST with 2.5% non-fat milk. The membranes were washed with PBS-T and incubated with appropriate horseradish peroxidase-conjugated secondary antibody at room temperature for 1 h; the signals were visualized by Chemiluminescent Peroxidase Substrate-1 or Super Signal West Femto Maximum Sensitivity Substrate (Thermo Fisher Scientific), using a Chemidoc^TM^ XRS detection system equipped with Image Lab Software (Bio-Rad). Densitometric analysis was performed by using ImageJ software (National Institute of Health, Bethesda, MD, United States). Protein expression level in the control sample was taken as 100%. Each result was expressed as a percentage of the value of the control sample. Each test was repeated three times.

### Lipid Accumulation Assay

Staining of intracellular neutral lipids was performed using Oil Red O. Stock solution was prepared by dissolving 0.14 g in 40 ml 2-propanol 100% (0.35% w/v). This stock solution was stored at room temperature for 2 h. The working solution was obtained by diluting three parts of stock solution with two parts of distilled water. Working solution was prepared freshly for each experiment and filtered immediately before use.

For qualitative analysis, HepG2 cells were seeded on coverslips (1.5 × 10^5^ cells/well) in a 24-well tissue culture plate and then treated with 10 mM sodium citrate for 24 h (except the untreated control); as a positive control of intracellular neutral lipid production, 2 mM oleic acid was used. The cells were washed twice with phosphate-buffered saline (PBS) and fixed with 4% paraformaldehyde in PBS for 30 min. Formalin was removed, and 2-propanol 60% was added for 5 min. 2-Propanol 60% was removed, and the cells were stained with Oil Red O working solution for 20 min at room temperature. After extensive washing with distilled water, the coverslips were removed from the wells and blotted to remove any excess water. Oil droplets were observed using fluorescence microscopy FLoid Cell^TM^ Imaging Station (Thermo Fisher Scientific) in cells with 4′,6-diamidino-2-phenylindole-stained nuclei (Fluoroshield^TM^ with DAPI, Sigma).

For quantitative analysis, HepG2 cells were seeded (1.5 × 10^5^ cells/well) in a clear 24-well microtiter plate and then incubated with 10 mM sodium citrate for 24 h (except the untreated control). After addition of Oil Red O working solution for 20 min at room temperature, the cells were treated with 2-propanol, and lipid accumulation was measured using a microplate reader (Multiskan^TM^ GO, Thermo Fisher Scientific) and absorbance was recorded at 510 nm. The results were presented as percentage of the control (cells untreated), defined as 100% of neutral lipid production. Each test was repeated three times in triplicate.

### Statistical Analysis

The data are presented as mean ± SD. Student’s *t*-test was performed pairwise to compare the control and the treated samples. Differences were considered significant whenever *p*-value < 0.05. Statistical analysis was performed using statistical GraphPad software.

## Results

### Effect of Citrate on Acetyl Group Availability in HepG2 Cells

**FIGURE 1:**
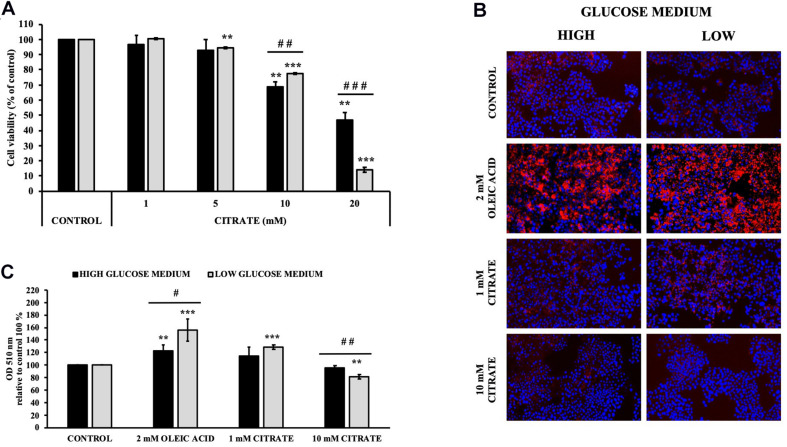
Effect of citrate on HepG2 cell viability and lipid droplet accumulation. **(A)** Viability of Cells grown at 25 mM (black bars) or 5 mM (gray bars) glucose were treated with different concentrations of citrate (1, 5, 10, and 20 mM) for 24 h. Data were expressed as percentage of the control group and presented as means ± SD of three replicates from three independent experiments. **p < 0.01, ***p < 0.001 cells treated with citrate vs untreated control; ^##^*p* < 0.01, ^###^*p* < 0.001 cells grown in low-glucose medium vs cells grown in high-glucose medium. **(B)** Microscope images of HepG2 cells grown at 25 mM (high) or 5 mM (low) glucose, treated with citrate 1 and 10 mM for 24 h and stained with Oil Red O to detect hepatic lipid droplets (red). The nuclei were stained with 4′,6-diamidino-2-phenylindole (blue). Cells without citrate are considered as control. Oleic acid treatment (2 mM) was used as positive control of intracellular neutral lipids accumulation. **(C)** Lipid droplets were quantified at 510 nm. Data were expressed as percentage of the control group and presented as means ± SD of three replicates from three independent experiments **p < 0.01, ***p < 0.001 treated cells vs control cells; ^#^*p* < 0.05, ^##^*p* < 0.01, cells grown in low-glucose medium (gray bars) vs cells grown in high-glucose medium (black bars).

The cell viability of HepG2 cells grown in 5 mM (low) and 25 mM (high) glucose medium and citrate was evaluated using the MTT assay. Incubation with sodium citrate for 24 h at concentrations ranging from 1 to 20 mM decreased the viability of HepG2 cells in a dose-dependent manner (Figure 1A). Citrate did not have a significant effect on cell viability at concentrations ≤5 mM. The IC_50_ values were approximately 17 and 13 mM for the cells grown in high- and low-glucose medium, respectively. At 20 mM concentration, the viability of cells was about 50 and 15% in high- and low-glucose media, respectively. In the cytosol, citrate is cleaved into acetyl-CoA and OAA by ACLY. Since citrate is a source of acetyl groups, its effect on lipid deposition and histone H4 acetylation has been evaluated.

Cells treated with 1 mM citrate showed a slight increase in lipid deposition as demonstrated by Oil Red O staining. Contrary to what was expected, no increase in lipid accumulation was observed with increasing citrate concentration, thus suggesting a lower availability of acetyl-CoA (Figures 1B,C).

**FIGURE 2:**
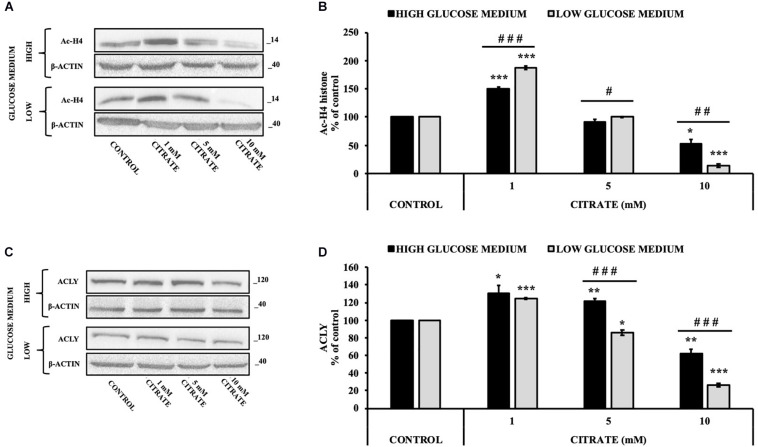
Effect of citrate on the acetylation of histone H4 and ACLY expression. Representative western blot **(A)** and **(B)** densitometric analysis of the immunoreactive bands of Ac-H4 in HepG2 cells grown at 25 and 5 mM glucose in the presence of 1, 5, and 10 mM of citrate for 24 h. The Ac-H4 levels were normalized with β-actin content. The results from three independent experiments are presented as a percentage of acetylated histone H4 compared with 100% of the control cells. Data are shown as mean ± standard deviation (SD); ^∗^*p* < 0.05, ***p < 0.001 cells treated with citrate vs control cells; ^#^ *p* < 0.05, ^##^*p* < 0.01, ^###^*p* < 0.001 cells grown in low-glucose medium vs cells grown in high-glucose medium. Representative western blot **(C)** and **(D)** densitometric analysis of the immunoreactive bands of ACLY protein in HepG2 cells grown in high- and low-glucose medium and in the presence of 1, 5, and 10 mM of citrate for 24 h. The protein levels were normalized with β-actin content. The results from three independent experiments are presented as a percentage of ACLY protein levels compared with 100% of the control cells. Data are shown as mean ± SD, *p < 0.05, **p < 0.01, ***p < 0.001 cells treated with citrate vs untreated control; ^###^*p* < 0.001 cells grown in low-glucose medium vs cells grown in high-glucose medium.

The cells were treated with various concentrations of citrate for 24 h, and the acetylation of histone H4 (Ac-H4 histone) was evaluated. In the presence of sodium citrate at 1 and 5 mM, an increase of Ac-H4 histone has been observed; however, at 10 mM citrate, the acetylated histone decreased both at high and low glucose concentrations (Figures 2A,B). These results prompted us to investigate the effect of citrate on ACLY expression (Figures 2C,D). The addition of citrate at 1 mM increased the ACLY expression when the cells were grown in both high- and low-glucose medium. However, the expression of ACLY was reduced at 5 mM citrate and decreased significantly at 10 mM citrate with a trend similar to that observed for the acetylation of H4 histone (Figure 2B), thus suggesting that the expression of ACLY is epigenetically controlled by acetylation of histone H4.

**FIGURE 3:**
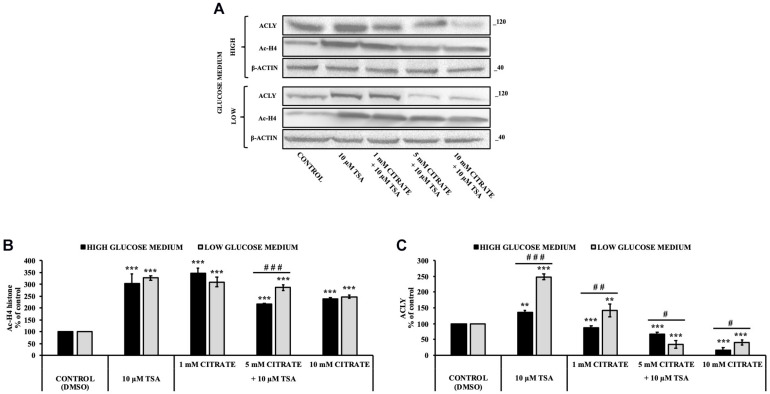
Effect of Trichostatin A (TSA) on the acetylation of histone H4 and ACLY expression in HepG2 cells treated with citrate. **(A)** Representative western blot of Ac-H4 histone and ACLY protein in HepG2 grown in high- and low-glucose medium and in the presence of 10 μM TSA alone and in combination with 1, 5, and 10 mM citrate for 24 h. Densitometric analysis of the immunoreactive bands of **(B)** Ac-H4 histone and **(C)** ACLY protein in three independent experiments. The levels of proteins were normalized with respect to the β-actin protein levels. The results from three independent experiments of cells grown at 25 and 5 mM glucose are presented as a percentage of ACLY and Ac-H4 compared with 100% of the control cells treated with 0.15% dimethyl sulfoxide (control). Data are shown as mean ± SD, **p < 0.01, ***p < 0.001 cells treated with TSA vs control cells or cells treated with TSA in combination with citrate vs TSA alone; ^#^*p* < 0.05, ^##^*p* < 0.01, ^###^*p* < 0.001 cells grown in low-glucose medium vs cells grown in high-glucose medium.

Since histone acetylation involves a balance between the activities of enzymes that catalyze histone acetylation (HAT) and deacetylation (HDAC), we evaluated the effect of citrate on the expression of ACLY and the acetylation of histone H4 in the presence of TSA, a deacetylation inhibitor. HepG2 cells were grown in high- and low-glucose medium and treated with 10 μM TSA alone and in combination with different concentrations of citrate (Figure 3A) for 24 h. The acetylated histone and the expression of ACLY increased after treatment with TSA, as compared to the control. With 1 mM citrate and TSA, the acetylation of histone H4 did not change significantly as compared to TSA alone (Figure 3B), whereas the expression of ACLY was considerably lowered (Figure 3C). Moreover, with TSA and citrate at a higher concentration, both acetylation of H4 and ACLY expression decreased in a dose-dependent manner. These results confirmed that citrate epigenetically modulates ACLY also in the presence of TSA, reducing the acetylation of histone H4.

### Effect of Citrate on the Viability, Acetylation of Histone H4, and ACYL Expression in Immortalized Human Hepatocytes

**FIGURE 4:**
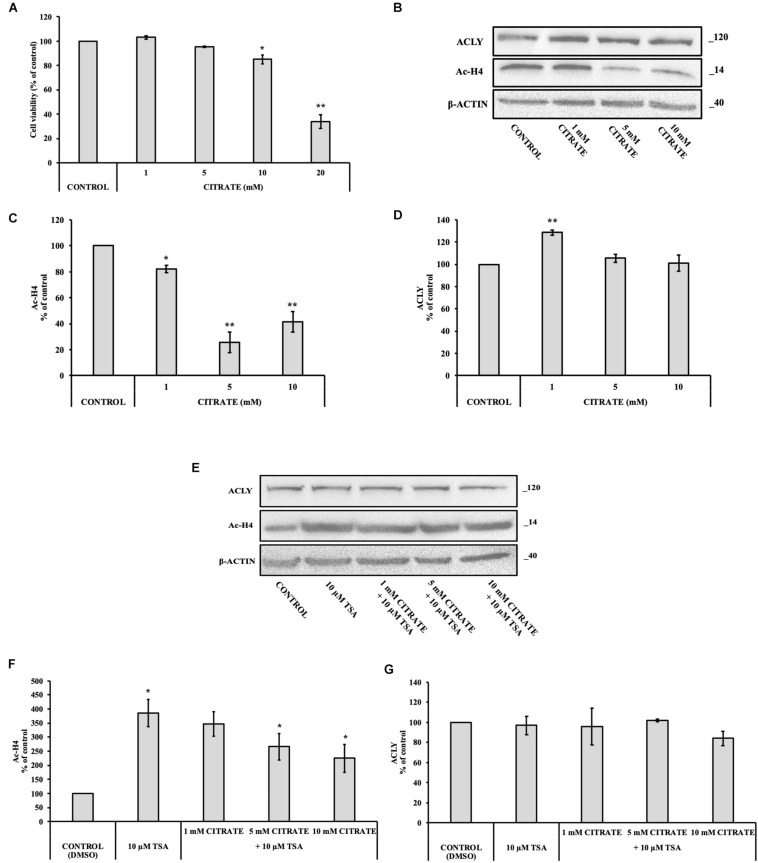
Effect of citrate on the acetylation of histone H4 and ACLY expression in immortalized human hepatic (IHH) cells. **(A)** Viability of cells treated with citrate at different concentrations (1, 5, 10, and 20 mM) for 24 h. Data were expressed as percentage of the control group and presented as means ± SD of three replicates from three independent experiments. *p < 0.05, **p < 0.01 cells treated with citrate vs untreated control. **(B)** Representative western blot of Ac-H4 histone and ACLY protein. Densitometric analysis of the immunoreactive bands performed in three independent experiments of **(C)** Ac-H4 histone and **(D)** ACLY protein. Protein levels were normalized with β-actin content. The results from three independent experiments are presented as a percentage of protein levels compared with 100% of the control cells. Data are shown as mean ± SD, *p < 0.05, **p < 0.01, cells treated with citrate vs untreated control. **(E)** Representative western blot of ACLY and Ac-H4 in IHH cells grown in the presence of 10 μM TSA alone and in combination with 1, 5, and 10 mM citrate for 24 h. Densitometric analysis of the immunoreactive bands performed in three independent experiments of **(F)** Ac-H4 histone and **(G)** ACLY. Protein levels were normalized with β-actin content. The results from three independent experiments are presented as a percentage of protein levels compared with 100% of the control cells. Data are shown as mean ± SD, *p < 0.05, cells treated with Trichostatin A (TSA) vs control cells or cells treated with TSA in combination with citrate vs TSA alone.

With the aim to verify the effect of citrate on normal cells, we performed some experiments on IHH cells. The administration of citrate at different concentrations, from 5 to 20 mM, significantly decreased the viability of IHH cells in a dose-dependent manner (Figure 4A). At 20 mM concentration of sodium citrate, the viability of IHH cells was reduced to about 40%.

We investigated the level of Ac-H4 histone and ACLY expression in cells grown with sodium citrate for 24 h at concentrations that ranged from 1 to 10 mM (Figure 4B). A significant decrease of Ac-H4 histone in up to 10 mM concentration of citrate has been observed (Figure 4C). On the contrary, the expression of ACLY did not change at 5 and 10 mM citrate; at 1 mM, it even increased as compared to the control (Figure 4D), thus suggesting that, in non-tumor cells, changes of acetylated histone level do not correspond to a change of ACLY expression, as instead shown by HepG2 cells.

In addition, we evaluated Ac-H4 histone and the expression of ACLY in IHH grown at different concentrations of citrate in combination with 10 μM TSA (Figure 4E). When cells are treated with the deacetylase inhibitor, citrate decreased the acetylation of histone H4 in a dose-dependent manner (Figure 4F). Once again, the expression of ACLY did not change (Figure 4G), thus confirming that, in IHH cells, citrate did not control ACLY expression by changing the acetylation of histone H4.

## Discussion

Citrate is an important metabolite in cellular energy metabolism, and it has multiple physiological and pathological functions. It acts as a regulator of glucose and lipid metabolism and controls the acetyl group availability. The anticancer properties of citrate were reported on different tumor cell lines; however, the effects of citrate to support cell proliferation have been also described (Zhang et al., 2009; Icard et al., 2012; Lincet et al., 2013; Xia et al., 2018).

In this study, we investigated the effects of different concentrations of extracellularly administered citrate, focusing on its main intracellular functions, i.e., to provide acetyl-CoA for the biosynthesis of fatty acids and histone acetylation. These two important processes are required by cancer cells to synthesize large quantities of lipids to build cell membranes and regulate gene expression, respectively.

Lipid deposition as well as acetylated histone H4 in HepG2 cells was increased when grown with 1 mM citrate and decreased with 10 mM citrate (Figures 1, 2). Since citrate is converted to acetyl-CoA and OAA by ACLY and acetyl-CoA is a substrate for both lipid synthesis and histone acetylation, one would expect an increase of both processes upon citrate supplementation. The unexpected inhibition of the ACLY activity in cells grown with high citrate concentration suggested us to further investigate the effects of citrate on ACLY expression. HepG2 cells grown in the presence of different citrate concentrations showed a clear correlation between acetylated H4 histone and ACLY protein expression. The same correlation was confirmed even in the presence of TSA, an inhibitor of histone deacetylases, thus suggesting that ACLY expression is partly controlled by histone H4 acetylation (Figure 3).

In order to verify if the unexpected result that we found after treatment with citrate 10 mM is due to a specific behavior of cancer cells, experiments were performed on IHH cells, which have a metabolism very similar to normal hepatocytes. The addition of 10 mM citrate decreased the acetylation of histone H4, which is similar to what was observed in HepG2 cells, suggesting that the regulation of histone acetylation occurs the same way in both tumor and non-tumor cells. In contrast, ACLY expression in IHH cells was not modified after citrate exposure, suggesting that, in this case, ACLY expression was not regulated by histone H4 acetylation (Figure 4). These results therefore suggest that the epigenetic regulation of ACLY in cancer cells is one way to adapt the metabolism for specific cellular requirements.

**FIGURE 5:**
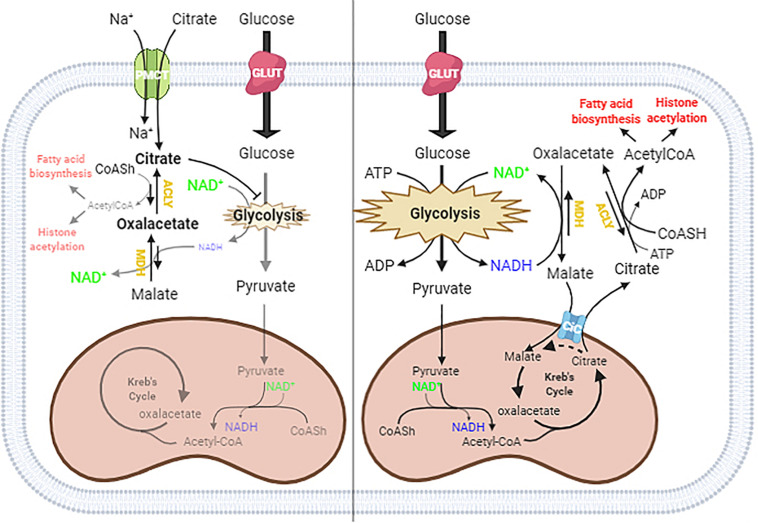
Proposed mechanism of citrate effect at a higher concentration. In the absence of exogenous citrate or with low citrate concentration (right panel), citrate formed in the TCA cycle moves to the cytoplasm through the citrate/malate exchanger and supplies acetyl groups for histone acetylation and lipid biosynthesis. Since citrate is converted by ATP citrate lyase (ACLY) in acetyl-CoA and oxaloacetate, which in turn goes back in the mitochondria after reduction to malate in exchange with citrate, the full process results in an outflow of acetyl groups. When citrate is administered at a high concentration (left panel), the oxaloacetate accumulates in the cytosol because of the lack of reducing equivalents needed to convert to malate, due to citrate itself acting as an inhibitor of glycolysis. The equilibrium balance of the reaction catalyzed by ACLY is shifted toward the reagents, lowering the availability of acetyl groups for lipid biosynthesis and histone acetylation.

In the schematic diagram shown in Figure 5, we propose a possible explanation of what might happen in cells under normal conditions and when citrate is given at a high concentration. In the cytoplasm, ACLY catalyzes the cleavage of citrate into acetyl-CoA and OAA. In normal conditions, citrate produced by mitochondria effluxes into the cytoplasm through the mitochondrial citrate carrier that exchanges the citrate with malate produced by the reduction of OAA: in this way, the acetylation process is strictly linked to the reduction of OAA to malate by the NADH produced by glycolysis.

The exogenous citrate enters the cells through the sodium-dependent transporter SLC13A5 (Costello and Franklin, 2016) and is cleaved in acetyl-CoA and OAA. In the presence of high exogenous citrate, we found decreased lipid deposition and histone acetylation, indicative of a decreased availability of acetyl-CoA, which suggested an inhibition of ACLY activity. The inhibition of ACLY may be due to several causes that we can consider individually or in combination, such as the strong inhibition of glycolysis, which leads to a decrease in NADH necessary for OAA reduction, OAA directly inhibiting ACLY, or simply a shift in the balance of the reaction catalyzed by ACLY due to the accumulation of OAA, which prevents the further synthesis of acetyl-CoA and OAA.

Considering the strong demand for acetyl-CoA in tumor cells but not for OAA, the administration of citrate at a high concentration to HepG2 cells determines an accumulation of OAA, which reduces the further synthesis of OAA and acetyl-CoA and therefore ACLY expression. For this reason, we suggest that the exogenous citrate would behave like a trojan horse that releases OAA in the cells, where it could exert its therapeutic effect also on hepatoma cells. Our hypothesis is also supported by what has already been reported on the antitumor potential of OAA due to its ability to inhibit glycolysis through the enhancement of oxidative phosphorylation (Kuang et al., 2018). Although further experiments are needed to define the molecular mechanism by which high concentrations of citrate inhibit ACLY, the results reported in this work shed light on some data in the literature regarding the use of citrate in the treatment of cancer. The most important discovery is undoubtedly the demonstration that high concentrations of citrate decrease the availability of acetyl-CoA, a key molecule both in the metabolism of sugars and lipids and in the control of gene transcription that lays the basis for the understanding of all the pathophysiological activities sensitive to citrate. Another important aspect that emerges from this work is that, although citrate is a metabolic intermediate, the exogenous citrate behaves differently from the endogenous citrate because the latter, due to the exchange mechanism with which the mitochondrial citrate transport operates, when it is cleaved by the ACLY, releases acetyl-CoA in the cytoplasm and reduced oxaloacetate to malate in the mitochondria which, in addition to feeding the mitochondrial activity, prevents the accumulation of oxaloacetate.

As citrate metabolism provides a connection between carbohydrate metabolism, fatty acid metabolism, and epigenetic reprogramming, its administration to higher concentrations compared to the physiological ones may be useful as anticancer drug for liver cancer.

## Data Availability Statement

The original contributions presented in the study are included in the article/supplementary material, further inquiries can be directed to the corresponding authors.

## Author Contributions

FB and AO conceived this work, designed the experiments, analyzed the data, and wrote the manuscript. AP performed the experiments. VA performed statistical analysis. PK critically read the manuscript. FB supervised the work. All authors contributed to the article and approved the submitted version.

## Conflict of Interest

The authors declare that the research was conducted in the absence of any commercial or financial relationships that could be construed as a potential conflict of interest.
